# Assessment and Prediction of Human Proteotypic Peptide Stability for Proteomics Quantification

**DOI:** 10.1021/acs.analchem.3c02269

**Published:** 2023-09-07

**Authors:** Cristina Chiva, Zahra Elhamraoui, Amanda Solé, Marc Serret, Mathias Wilhelm, Eduard Sabidó

**Affiliations:** † Centre for Genomics Regulation, Barcelona Institute of Science and Technology (BIST), Barcelona 08003, Spain; ‡ Universitat Pompeu Fabra, Barcelona 08003, Spain; § Technical University of Munich, Freising 85354, Germany

## Abstract

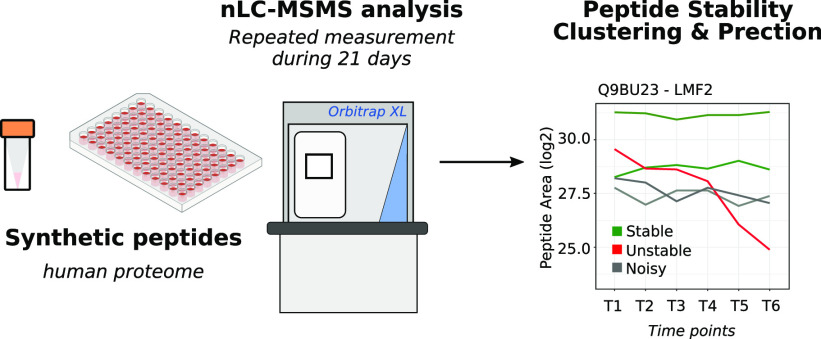

Mass spectrometry coupled to liquid chromatography is one of the most powerful technologies for proteome quantification in biomedical samples. In peptide-centric workflows, protein mixtures are enzymatically digested to peptides prior their analysis. However, proteome-wide quantification studies rarely identify all potential peptides for any given protein, and targeted proteomics experiments focus on a set of peptides for the proteins of interest. Consequently, proteomics relies on the use of a limited subset of all possible peptides as proxies for protein quantitation. In this work, we evaluated the stability of the human proteotypic peptides during 21 days and trained a deep learning model to predict peptide stability directly from tryptic sequences, which together constitute a resource of broad interest to prioritize and select peptides in proteome quantification experiments.

Mass spectrometry coupled to liquid chromatography is one of the most powerful technologies for proteome quantification in biomedical samples.^1^ In peptide-centric workflows, protein mixtures are enzymatically digested to peptides prior their analysis.^2^ Experimental spectra are then used for peptide identification, and extracted peptide areas, peak heights, or spectral counts are used to infer protein quantities.^3−5^ However, proteome-wide quantification studies rarely identify all potential peptides for any given protein, and targeted proteomics experiments focus on a set of peptides for the proteins of interest. Consequently, proteomics relies on the use of a limited subset of all possible peptides as proxies for protein quantitation. As only some peptides are used to infer protein abundances, the selected peptides become relevant to achieve accurate and precise protein quantification.

Several targeted and nontargeted proteomics studies have evaluated the quantitative response of tryptic peptides and have described rules to guide peptide selection for protein quantification.^6−8^ We and others have evaluated the influence of the digestion technique, protease, missed cleavages, and amino acidic composition in protein quantitation^9,10^ and showed the importance of experimental data to assess the quantitative behavior of peptides.^11^ In addition, various tools were developed that use previous experimental data, peptide amino acidic composition, and their physiochemical properties to derive suitability scores and thus suggest the best candidate peptides for quantitative proteomics.^8,12−15^ Beyond the effect of tryptic digestion and missed cleavages, short- and midterm peptide stability can have a profound effect on quantification, especially in large proteomics experiments that expand for several days or weeks. Despite its importance in other fields,^16^ to date only few studies have investigated peptide stability, either in the context of handling and storage of liquid biopsies,^17,18^ or in the development of specific targeted proteomics assays.^19−21^ Here, we expanded this knowledge by assessing the stability of the human proteotypic peptides under autosampler conditions for a period of 21 days, and we used this knowledge to train a deep learning model that predicts the stability for new tryptic peptide sequences.

We initially assessed the stability of the human proteotypic peptide set of the ProteomeTools collection,^22^ containing 124,875 peptides and mapping to 15,990 human Uniprot/SwissProt annotated genes (Table ST1). Synthetic peptide pools consisted of approximately 1000 peptides (∼5 pmol each) disolved in water with 0.1% formic acid and kept at 4 °C in polypropylene vials during the entire experiment. Each pool was analyzed by data-dependent acquisition LC-MSMS once every 3.5 days, with a total of 6 time points within a period of 21 days (Figure 1A). Acquired data were analyzed using MaxQuant v1.6.0,^23^ with match between runs among the six independent injections of each peptide pool. Peptides were identified at FDR < 5% given the known composition of each peptide pool, and peptide areas were extracted for peptide relative quantification (Table ST2). On average, peptide identifications that surpassed 85% of the total analyzed peptides were peptide identifications, with pools reaching up to 95% identification success rate, and most of the identifications being based on fragmentation spectra evidence (Figure S1A, B). Profiles with less than three quantitative points were discarded, and the remaining missing values in each profile were imputed as the average of their neighboring temporal quantitative values. Quantitative temporal profiles comprising 6 injections within 21 days (822 injections) were obtained for 101,903 proteotypic peptides in 137 pools, making a total of over half a million data points. Peptide intensities were normalized within each pool based on median equalization (Figure S1C).

**Figure 1:**
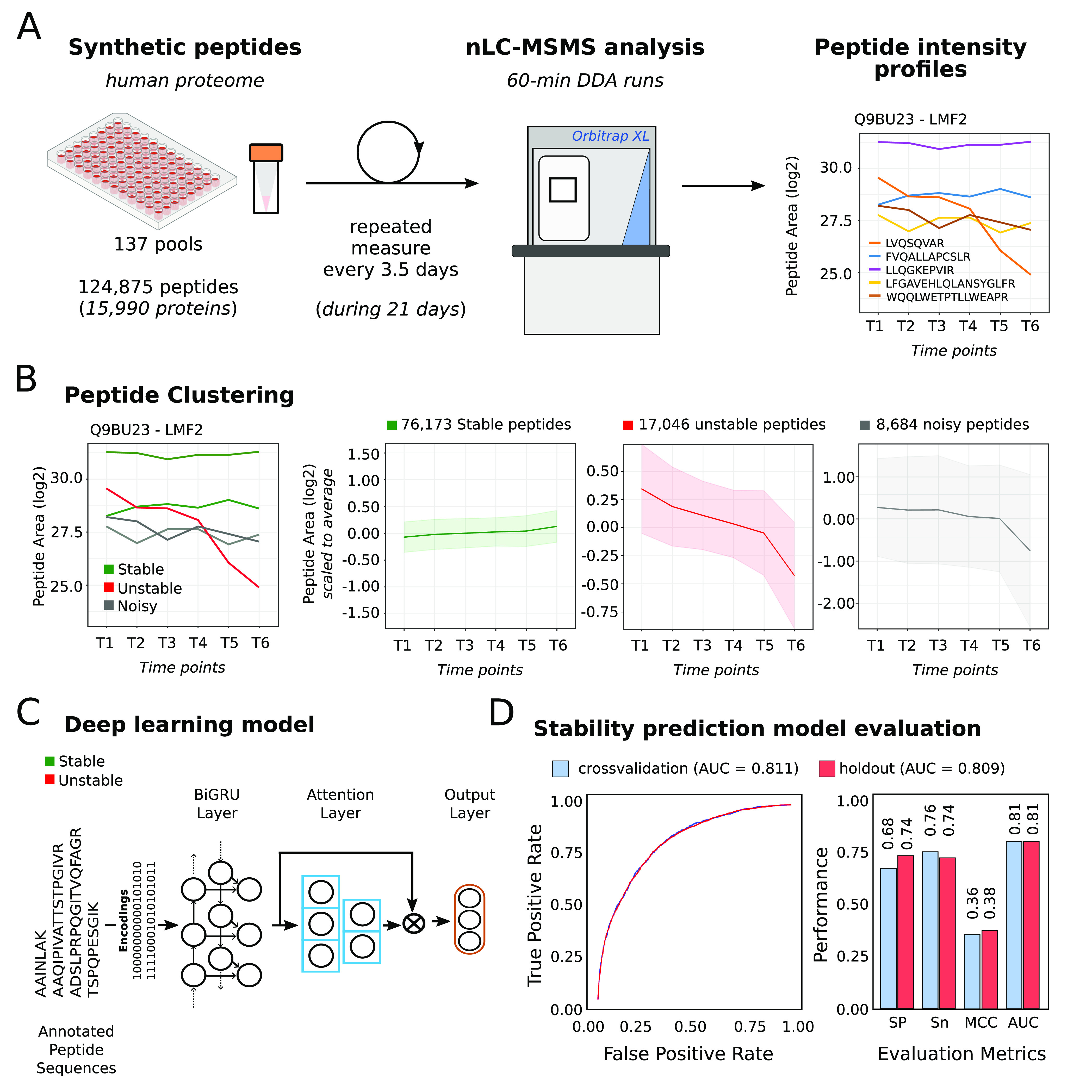
(A) Schematic representation of the experimental workflow for peptide stability assessment. (B) Peptide stability profile clusters obtained from experimental data. (C) Schematic representation of the deep learning model trained in this work to predict peptide stability. (D) Sensitivity, specificity, accuracy, Matthews correlation coefficient, and average receiver operating characteristic (ROC) for the deep learning model.

Peptide profiles were used to assess the stability of each peptide and distinguish between stable and unstable peptides. We used a deep embedding clustering (DEC)^24^ algorithm, an unsupervised deep learning model that combines deep embedding and k-means clustering (see Supporting Information). The peptide abundance profiles were grouped based on pattern similarity, resulting in three subgroups containing 76,173 (74.75%), 17,046 (16.72%), and 8,684 (8.52%) peptide sequences. Based on their average profile, we labeled the obtained clusters as “*stable*” (*cluster* #3) and “*unstable*” (*cluster* #2) peptide sequences and “*noisy data*” (*cluster* #1), respectively (Figure 1B, Table ST2). Despite peptide stability being affected by specific matrix effects, plastic and glassware, and storage conditions, our results demonstrate that most proteotypic peptides from the human proteome are stable throughout a period of 21 days in specific measuring conditions that are common in many proteomics laboratories.

Next, we used these labeled experimental data to build a deep learning model that can learn and predict peptide stability for any tryptic peptide of interest using only its amino acid sequence. A total of 77,338 peptide experimental stability profiles were used as training set, whereas 13,649 peptide profiles were set as an independent test data set to evaluate the model performance. We used a deep learning model with a hybrid architecture that included two Bidirectional Gated Recurrent Unit (BiGRU) layers and an attention mechanism layer (Figure 1C). To assess the performance of our model, we used 5-fold cross-validation and a holdout set. Due to the imbalanced nature of our data set, we used AUC as our evaluation metric. Our model obtained an AUC of 0.809 on the holdout set and 0.811 during cross-validation, demonstrating its robustness in peptide stability prediction (Figure 1D). Finally, we built an online web server, hosting both the experimental stability data for the measured proteotypic peptides and the model to predict peptide stability for new tryptic peptide sequences (http://peptidestability.crg.eu). The latter is limited to peptide sequences of length below 20 amino acids with no chemical or post-translational modifications.

Overall, we evaluated the stability of the human proteotypic peptides during 21 days and trained a deep learning model to predict peptide stability directly from tryptic sequences, which together constitute a resource of broad interest within the community to prioritize and select peptides in proteome quantification experiments.

## Data Availability

The mass spectrometry proteomics data have been deposited at the ProteomeXChange Consortium via the PRIDE repository with identifier PXD025766,^25^ and the model source code can be found at https://github.com/proteomicsunitcrg/peptide-stability.

## Supporting Information Available

Figure S1: Heatmap representing the number of peptides identified in each pool; number of time points with valid identifications; peptide areas distribution before and after median normalization; peptide abundance profiles along time (T1–T6) for protein MICAL3; peptide abundance profiles along time (T1–T6) for protein LMF2; number of amino acid containing peptides that are stable, unstable, or noisy, either as total counts or relative frequencies. Supplementry methods. (PDF)
Table ST1: (A) List of human proteotypic peptides from the ProteomeTools collection measured in this study and (B) list of quality control peptides included in each peptide pool of the ProteomeTools collection (XLSX)
Table ST2: Identification and quantification of peptide stability profiles within 21 days (3.5 days per time point) (XLSX)
