# Supplementary material for: Assessment and Prediction of Human Proteotypic Peptide Stability for Proteomics Quantification
Source: Anal Chem. 2023 Sep 7;95(37):13746–9. doi: 10.1021/acs.analchem.3c02269 (PMC10515110; doi:10.1021/acs.analchem.3c02269)
Supplement: Supplementary file 1 — ac3c02269_si_001.pdf [file ac3c02269_si_001.pdf]

Supporting Information

Assessment and Prediction of Human Proteotypic Peptide Stability for Proteomics Quantification

Cristina Chiva[1,2,$], Zahra Elhamraoui[1,2,$], Amanda Solé[1,2], Marc Serret[1,2], Mathias Wilhelm[3], Eduard Sabidó[1,2,*]

[1]*Center for Genomics Regulation, Barcelona Institute of Science and Technology (BIST), Barcelona, Spain*

[2]*Universitat Pompeu Fabra, Barcelona, Spain*

[3]*Technical University of Munich, Freising, Germany*

$ Equal contribution

* Corresponding author: Eduard Sabidó; Proteomics Unit, Center for Genomics Regulation, Barcelona Institute of Science and Technology (BIST), Universitat Pompeu Fabra, Barcelona, Spain; Dr. Aiguader 88; 08003 Barcelona. Email: eduard.sabido@crg.cat

Table of Contents

Table ST1: A) List of human proteotypic peptides from the ProteomeTools collection measured in this study; B) List of quality control peptides included in each peptide pool of the ProteomeTools collection.

Table ST2: Identification and quantification of peptide stability profiles within 21 days (3.5 days per time point).

Figure S1: A) Heatmap representing the number of peptides identified in each pool; B) Number of time points with valid identifications; C) Peptide areas distribution before and after median normalization; D) Peptide abundance profiles along time (T1-T6) for protein MICAL3; E) Peptide abundance profiles along time (T1-T6) for protein LMF2; F-G) Number of amino acid containing peptides that are stable, unstable or noisy, either as total counts (F) or relative frequencies (G).

Supplementary Methods

Supplementary Figures

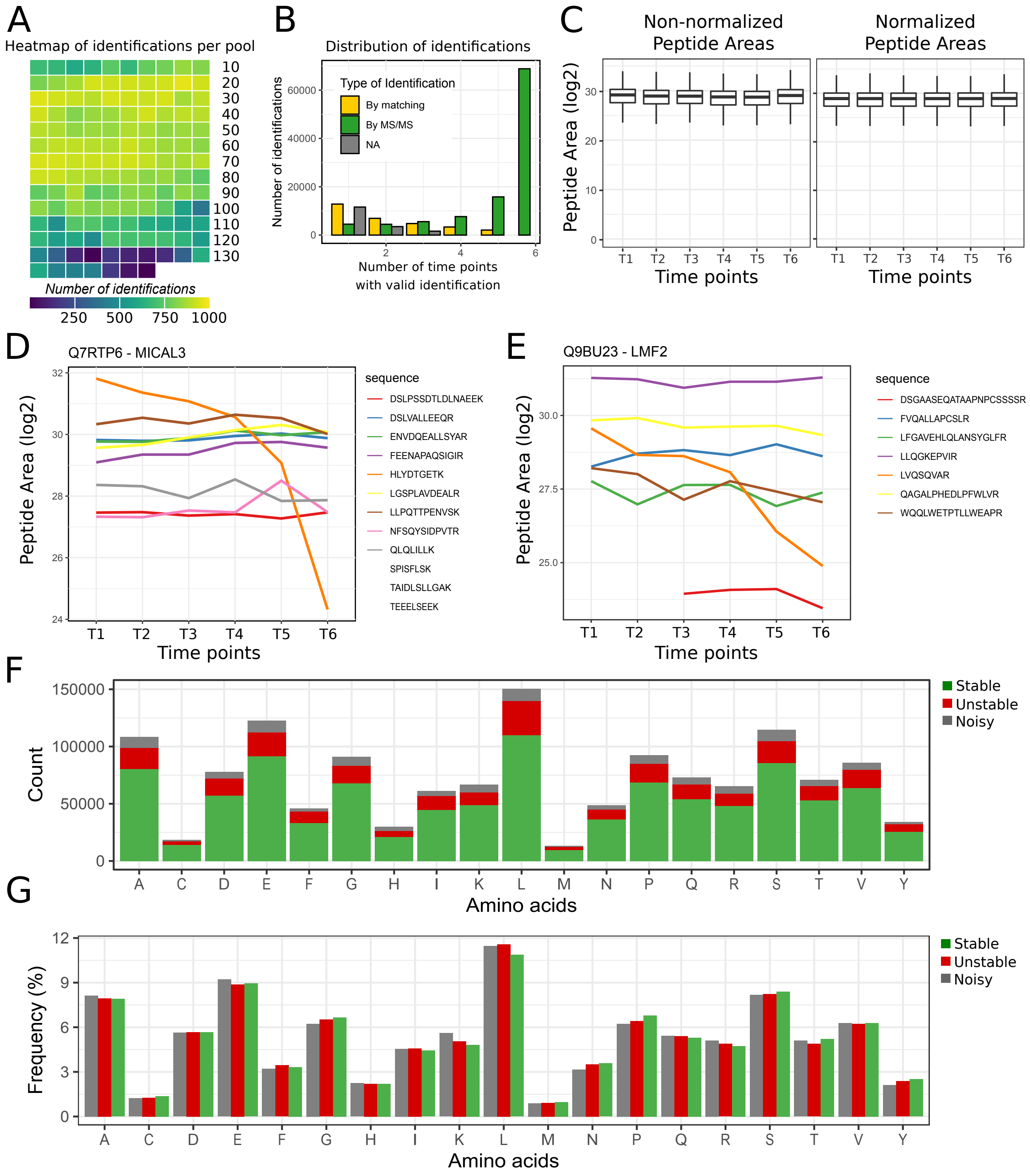

Figure S1: A) Heatmap representing the number of peptides identified in each pool; B) Number of time points with valid identifications; C) Peptide areas distribution before and after median normalization; D) Peptide abundance profiles along time (T1-T6) for protein MICAL3; E) Peptide abundance profiles along time (T1-T6) for protein LMF2; F-G) Number of amino acid containing peptides that are stable, unstable or noisy, either as total counts (F) or relative frequencies (G).

Supplementary Tables

Table ST1: A) List of human proteotypic peptides from the ProteomeTools collection measured in this study; B) List of quality control peptides included in each peptide pool of the ProteomeTools collection.

Table ST2: Identification and quantification of peptide stability profiles within 21 days (3.5 days per time point).

Supplementary Methods

Experimental peptide stability assessment

The human proteotypic peptide set from the ProteomeTools collection[1] was build using information from prior mass-spectrometric evidence available in ProteomicsDB,[1,2] resulting in a peptide collection that contained 124,875 peptides covering 15,855 human Uniprot/SwissProt annotated genes. These synthetic peptides were mixed in pools of approximately 1000 peptides using an estimated amount of 5 pmol of each peptide plus five quality control peptides that were synthesized along with every peptide pool. Moreover, each pool was spiked with two retention time (RT) standards, 5 pmol from an extended PROCAL (JPT iRT)[3] standard mix consisting of 66 peptides with non-naturally occurring peptides, and 2.5 pmol from a set of 15 ^{13}C,^{15}N-labelled peptide standards (Pierce, Thermo Fisher Scientific) (**Table ST1B**). Each peptide pool was dissolved in water + 0.1% formic acid and kept at 4 ºC in polypropylene vials (C4011-13 11mm PP VIAL CRIMP/SNAP 250 µL, Thermo Fisher Scientific) during the entire experiment. Vial caps were replaced for new ones after every injection (CC-C4011-540OR Cap, Snap, 11mm, Orange, PTFE/Silicone, 0.04", Thermo Fisher Scientific). Each pool was analyzed once every 3.5 days, with a total of 6 time points within a period of 21 days.

Samples were analyzed by shotgun data-dependent acquisition LC-MSMS with an EASY-nLC 300 (Proxeon) and a LTQ-Orbitrap XL (Thermo Fisher Scientific) mass spectrometer. Peptides pools were loaded onto the 2-cm Nano Trap column with an inner diameter of 100 µm packed with C18 particles of 5 µm particle size (Thermo Fisher Scientific) and were separated by reversed-phase chromatography using a 12-cm column with an inner diameter of 75 µm, packed with 3 µm C18 particles (Nikkyo Technos Co., Ltd. Japan). Chromatographic gradients started at 97% buffer A and 3% buffer B with a flow rate of 300 nl/min for 4 minutes and gradually increased to 35% buffer B and 65% buffer A for 30 min. The column was washed with 10 % buffer A and 90% buffer B for 10 min after each analysis. Buffer A: 0.1% formic acid in water. Buffer B: 0.1% formic acid in acetonitrile.

The mass spectrometer was operated in positive ionization mode with nanospray voltage set at 2.25 kV and source temperature at 200°C. Ultramark 1621 for the was used for external calibration of the FT mass analyzer prior the analyses, and an internal calibration was performed using the background polysiloxane ion signal at m/z 445.1200. The acquisition was performed in data-dependent acquisition (DDA) mode and full MS scans with 1 micro scans at resolution of 60,000 were used over a mass range of m/z 350-1500 with detection in the Orbitrap mass analyzer. Auto gain control (AGC) was set to 2E5, dynamic exclusion (60 seconds) and charge state filtering disqualifying singly charged peptides was activated. In each cycle of DDA analysis, following each survey scan, the top ten most intense ions with multiple charged ions above a threshold ion count of 5000 were selected for fragmentation. Fragment ion spectra were produced via collision-induced dissociation (CID) at normalized collision energy of 35% and they were acquired in the ion trap mass analyzer. For MS2 spectra AGC

was set to 1E4, isolation window of 2.0 m/z, activation time of 30 ms and maximum injection time of 100 ms was used. All data were acquired with Xcalibur software v2.1.

Acquired data were analyzed using MaxQuant v1.6.0,[4,5] with match between runs among the six independent injections of each peptide pool. A custom fasta file was created for containing the all the peptide sequences within the human proteotypic peptide set plus the retention time and quality control reference peptides aforementioned. For peptide identification a precursor ion mass tolerance of 20 ppm was used for the first search and 4.5 ppm was used for the main search was used for MS1 level. Trypsin was chosen as enzyme and up to two missed cleavages were allowed. The fragment ion mass tolerance was set to 0.5 Da for MS2 spectra. Oxidation of methionine was used as variable modifications whereas carbamidomethylation on cysteines was set as a fixed modification. False discovery rate (FDR) in peptide identification was set to a maximum of 5%.Peptides were identified at FDR<5%, and peptide areas were extracted for peptide relative quantification.

The mass spectrometry proteomics data have been deposited at the ProteomeXChange Consortium via the PRIDE repository with identifier PXD035198.[6]

Peptide stability clustering model with TensorFlow

Peptide intensities were log-transformed within each pool. Profiles with less than three quantitative points were discarded, and the remaining missing values in each profile were imputed as the average of their neighboring temporal quantitative values. The intensity values of each peptide were centered on the intensity of day 0, which was assigned as the starting intensity for each temporal profile. Duplicated peptide sequences were removed by prioritizing profiles with a smaller number of missing values, more MS/MS identification evidence, and higher intensity values. We used the elbow in the k-means algorithm to estimate the number of clusters, where 2 and 3 are shown to be optimal, 3 clusters are chosen in this article.[7] The full experimental data defining the profile of each peptide was fed into a Deep Embedded Clustering (DEC) model.[8] We implemented the architecture following the same network architecture of the initial paper, the dimension of the input data is the input size. The encoder and decoder are sequential models, all linear layers except the last ones used sigmoid activation. All layers are densely connected. The initialization of the encoder and decoder's weights was done using a uniform distribution. Given that input is the input data dimension, the autoencoder network dimensions were set to (input, 10,10,6), the model used sigmoid as an activation function, Adam optimizer,[9] and a learning rate of 0.0001. The encoder layers are then connected to the clustering layer,[10] it computes a soft assignment between the embedded points and the cluster centroids, and then assigns an estimated class to each of the data samples in such a way that it can be refined iteratively. In the DEC training, Stochastic Gradient Descent (SGD)[11] is used to simultaneously optimize the cluster centers and the encoder parameters (feature representations) with a momentum of 0.4, while minimizing the mean square error loss between the DEC output and the auxiliary target distribution. The deep autoencoder is finetuned manually for 5 epochs. The batch size is set to 32, with a

constant learning rate of 0.0001. We used k-means with 20 restarts, to initialize centroids in the same way described in the original study. The training is stopped when less than the tolerance % of points change cluster assignment between two consecutive epochs. The convergence threshold is set to tolerance = 0.0001. Furthermore, the pre-trained autoencoder and K-means model are used to define a new model that takes the preprocessed dataset as input and outputs both the predicted clustering classes and decoded input data records. The implementation was done using Python TensorFlow, Keras, and Scikit-learn.

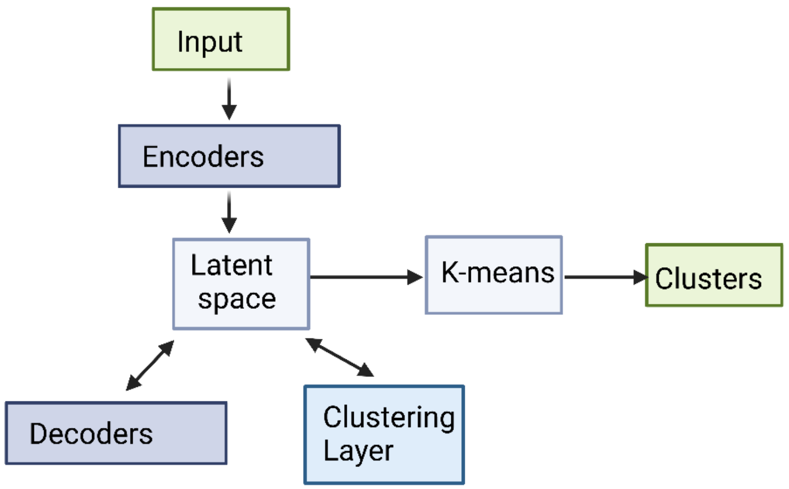

Scheme 1: DEC with K-means architecture, the model consists of 3 decode layers, 3 encoder layers, a clustering layer, and K-means clustering.

Peptide stability prediction model

Model Architecture

Given a peptide sequence, the amino acids are replaced with predefined unique numbers ranging from 1 to 21. Since the peptides are of different lengths, if the peptide length is less than the maximum value, the sequence is padded by adding zeros to the vectors. An embedding layer that maps the amino acids to dense vectors of 128 dimensions was then implemented. The peptide stability prediction model contains two Bidirectional Gated Recurrent Unit (Bi-GRU)[12] layers and an attention layer based on the Hierarchical attention networks,[13] all with dropouts and a spacial dropout.[14] The recurrent layers were used to extract important position information from peptide sequences, with a TanH[15] activation function, a dimension of 126, and L2 regularization.[16] The attention layer is used to calculate dynamic adaptive weights based on probability distribution. Finally, a sigmoid activation function was implemented into a dense layer. We used a binary cross-entropy loss, an Adam optimizer, and a binary accuracy to compile the model. Implementation was done with Python, Keras, and TensorFlow, prediction model is also available at https://github.com/proteomicsunitcrg/peptide-stability.

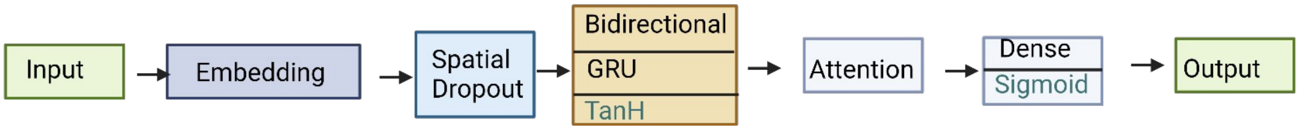

Scheme 2: Model Architecture of peptide stability predictions model, the input is a peptide sequence, and the model contains an embedding layer, Spatial Dropout, BiGRU, and an attention layer.

Training

The whole dataset contains 93,219 peptide sequences, where we used Python Scikit-learn to split our data set into 85% training and 15% holdout dataset, each peptide sequence is present only in either the training dataset (stable peptides, unstable peptides) or the holdout dataset(stable peptides, unstable peptides), full data available at https://github.com/proteomicsunitcrg/peptide-stability. The model hyperparameters were optimized manually, during the training, we used a batch size of 32, and a learning rate of 1e-03. TensorFlow function ReduceLROnPlateau was used to reduce the learning rate when no improvement was seen in 5 epochs, with a minimum learning rate of 1e-12. We also used an early stopping with a waiting of 5 epochs, the training showed no improvement after 25 epochs. We performed 10-fold cross-validation, where the training dataset is split into 10 folds, 9 folds are used for training while the remaining fold is used as a test, we built 10 models and randomly selected one as our final model. Then we evaluated the performance of our model on the holdout dataset. We plot the model loss history on the training and validation datasets.

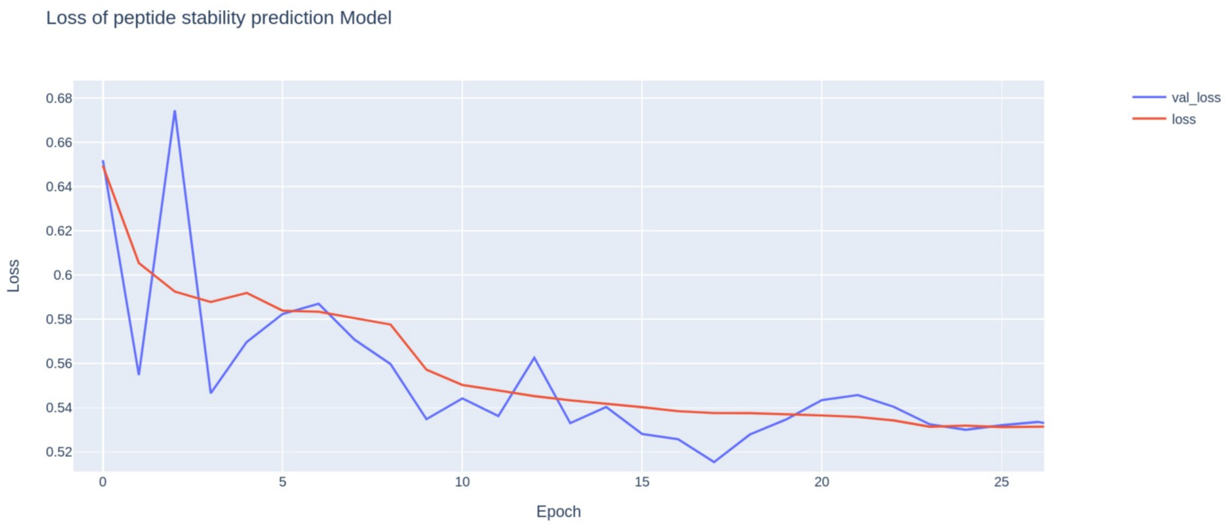

Scheme 3: Line plots of the training and validation loss values over several training epochs

After manually extracting features using amino acid composition, we tested multiple classical machine learning models, such as random forest,[17] XG-boost[18] and Adaboost classifier.[19] On the holdout dataset, our model had a good trade-off between Sensitivity (Sn) and Specificity (Sp) compared to both XG-boost and Random Forest, the model also outperformed in both the F1 score and the Area Under Curve (AUC), results are shown in the next table.

Scheme 4: Performance comparison of Random Forest, XG-boost, Adaboost, and our model

Model	Sp	Sn	MCC	F1-score	AUC
Random Forest	0.98	0.11	0.20	0.19	0.74
XG-boost	0.96	0.20	0.27	0.30	0.76
Adaboost	0.99	0.04	0.11	0.08	0.71
**Our Model**	**0.74**	**0.73**	**0.38**	**0.50**	**0.81**

Bibliography

(1) Zolg, D. P.; Wilhelm, M.; Schnatbaum, K.; Zerweck, J.; Knaute, T.; Delanghe, B.; Bailey, D. J.; Gessulat, S.; Ehrlich, H.-C.; Weininger, M.; Yu, P.; Schlegl, J.; Kramer, K.; Schmidt, T.; Kusebauch, U.; Deutsch, E. W.; Aebersold, R.; Moritz, R. L.; Wenschuh, H.; Moehring, T.; Aiche, S.; Huhmer, A.; Reimer, U.; Kuster, B. Building ProteomeTools Based on a Complete Synthetic Human Proteome. *Nat. Methods* **2017**, *14* (3), 259–262. https://doi.org/10.1038/nmeth.4153.

(2) Wilhelm, M.; Schlegl, J.; Hahne, H.; Gholami, A. M.; Lieberenz, M.; Savitski, M. M.; Ziegler, E.; Butzmann, L.; Gessulat, S.; Marx, H.; Mathieson, T.; Lemeer, S.; Schnatbaum, K.; Reimer, U.; Wenschuh, H.; Mollenhauer, M.; Slotta-Huspenina, J.; Boese, J.-H.; Bantscheff, M.; Gerstmair, A.; Faerber, F.; Kuster, B. Mass-Spectrometry-Based Draft of the Human Proteome. *Nature* **2014**, *509* (7502), 582–587. https://doi.org/10.1038/nature13319.

(3) Zolg, D. P.; Wilhelm, M.; Yu, P.; Knaute, T.; Zerweck, J.; Wenschuh, H.; Reimer, U.; Schnatbaum, K.; Kuster, B. PROCAL: A Set of 40 Peptide Standards for Retention Time Indexing, Column Performance Monitoring, and Collision Energy Calibration. *Proteomics* **2017**, *17* (21). https://doi.org/10.1002/pmic.201700263.

(4) Cox, J.; Mann, M. MaxQuant Enables High Peptide Identification Rates, Individualized p.p.b.-Range Mass Accuracies and Proteome-Wide Protein Quantification. *Nat. Biotechnol.* **2008**, *26* (12), 1367–1372. https://doi.org/10.1038/nbt.1511.

(5) Tyanova, S.; Temu, T.; Cox, J. The MaxQuant Computational Platform for Mass Spectrometry-Based Shotgun Proteomics. *Nat. Protoc.* **2016**, *11* (12), 2301–2319. https://doi.org/10.1038/nprot.2016.136.

(6) Vizcaíno, J. A.; Deutsch, E. W.; Wang, R.; Csordas, A.; Reisinger, F.; Ríos, D.; Dianes, J. A.; Sun, Z.; Farrah, T.; Bandeira, N.; Binz, P.-A.; Xenarios, I.; Eisenacher, M.; Mayer, G.; Gatto, L.; Campos, A.; Chalkley, R. J.; Kraus, H.-J.; Albar, J. P.; Martinez-Bartolomé, S.; Apweiler, R.; Omenn, G. S.; Martens, L.; Jones, A. R.; Hermjakob, H. ProteomeXchange Provides Globally Coordinated Proteomics Data Submission and Dissemination. *Nat. Biotechnol.* **2014**, *32* (3), 223–226. https://doi.org/10.1038/nbt.2839.

(7) Likas, A.; Vlassis, N.; J. Verbeek, J. The Global K-Means Clustering Algorithm. *Pattern Recognit.* **2003**, *36* (2), 451–461. https://doi.org/10.1016/S0031-3203(02)00060-2.

(8) Xie, J.; Girshick, R.; Farhadi, A. Unsupervised Deep Embedding for Clustering Analysis. arXiv May 24, 2016. https://doi.org/10.48550/arXiv.1511.06335.

(9) Kingma, D. P.; Ba, J. Adam: A Method for Stochastic Optimization. arXiv January 29, 2017. https://doi.org/10.48550/arXiv.1412.6980.

(10) Guo, X.; Liu, X.; Zhu, E.; Yin, J. Deep Clustering with Convolutional Autoencoders. In *Neural Information Processing*; Liu, D., Xie, S., Li, Y., Zhao, D., El-Alfy, E.-S. M., Eds.; Lecture Notes in Computer Science; Springer

International Publishing: Cham, 2017; pp 373–382. https://doi.org/10.1007/978-3-319-70096-0_39.
(11) Ruder, S. An Overview of Gradient Descent Optimization Algorithms. arXiv June 15, 2017. https://doi.org/10.48550/arXiv.1609.04747.
(12) Rana, R. Gated Recurrent Unit (GRU) for Emotion Classification from Noisy Speech. arXiv December 12, 2016. https://doi.org/10.48550/arXiv.1612.07778.
(13) Yang, Z.; Yang, D.; Dyer, C.; He, X.; Smola, A.; Hovy, E. Hierarchical Attention Networks for Document Classification. In *Proceedings of the 2016 Conference of the North American Chapter of the Association for Computational Linguistics: Human Language Technologies*; Association for Computational Linguistics: San Diego, California, 2016; pp 1480–1489. https://doi.org/10.18653/v1/N16-1174.
(14) Tompson, J.; Goroshin, R.; Jain, A.; LeCun, Y.; Bregler, C. Efficient Object Localization Using Convolutional Networks. arXiv June 9, 2015. https://doi.org/10.48550/arXiv.1411.4280.
(15) Namin, A. H.; Leboeuf, K.; Muscedere, R.; Wu, H.; Ahmadi, M. Efficient Hardware Implementation of the Hyperbolic Tangent Sigmoid Function. In *2009 IEEE International Symposium on Circuits and Systems*; 2009; pp 2117–2120. https://doi.org/10.1109/ISCAS.2009.5118213.
(16) Cortes, C.; Mohri, M.; Rostamizadeh, A. L2 Regularization for Learning Kernels. arXiv May 9, 2012. https://doi.org/10.48550/arXiv.1205.2653.
(17) Breiman, L. Random Forests. *Mach. Learn.* **2001**, *45* (1), 5–32. https://doi.org/10.1023/A:1010933404324.
(18) *XGBoost | Proceedings of the 22nd ACM SIGKDD International Conference on Knowledge Discovery and Data Mining*. https://dl.acm.org/doi/10.1145/2939672.2939785 (accessed 2023-04-25).
(19) Schapire, R. E. Explaining AdaBoost. In *Empirical Inference: Festschrift in Honor of Vladimir N. Vapnik*; Schölkopf, B., Luo, Z., Vovk, V., Eds.; Springer: Berlin, Heidelberg, 2013; pp 37–52. https://doi.org/10.1007/978-3-642-41136-6_5.
